# *Salvia miltiorrhiza* Root Extract for Men with Lower Urinary Tract Symptoms: A Multicenter, Randomized, Double-Blind, Placebo-Controlled Trial

**DOI:** 10.3390/nu17010024

**Published:** 2024-12-25

**Authors:** Dongho Shin, Hyong Woo Moon, Woong Jin Bae, U-Syn Ha, Young Ho Park, Eun Joo Lee, Du Geon Moon, Sae Woong Kim

**Affiliations:** 1 Department of Urology, College of Medicine, The Catholic University of Korea, Seoul 06591, Republic of Korea; eds8813@naver.com (D.S.);; 2 Healthism Corporation, Suwon 16229, Republic of Korea; 3 Department of Urology, College of Medicine, Korea University, Seoul 08308, Republic of Korea; 4 Green Medicine Co., Busan 48307, Republic of Korea

**Keywords:** lower urinary tract symptoms, International Prostate Symptom Score, *Salvia miltiorrhiza*

## Abstract

**Background:** The prevalence of urological diseases increases with age, and lower urinary tract symptoms (LUTSs) are the most common problem. Natural compounds with minimal side effects for the improvement in LUTSs are of ongoing interest. *Salvia miltiorrhiza root* extract (SAGX) has shown potential in preclinical studies for its effects on LUTSs. **Objectives:** This multicenter, randomized, double-blind, placebo-controlled study aimed to evaluate the efficacy and safety of SAGX in men with lower urinary tract symptoms (LUTSs) over a 12-week period. **Methods:** A total of 136 subjects were randomized to receive either 400 mg or 800 mg of SAGX or a placebo daily, orally. The primary outcome was the change in the International Prostate Symptom Score (IPSS). Secondary outcomes included changes in prostate-specific antigen (PSA), testosterone levels, urinary flow rate, residual urine volume, and erectile function as measured by the International Index of Erectile Function (IIEF). **Results:** Both SAGX intake groups showed statistically significant improvements in total IPSS scores and several secondary outcomes compared with the placebo group. Notable improvements were observed in symptoms of incomplete emptying, frequency, intermittency, weak stream, urgency, nocturia, and quality of life scores. Erectile function, as assessed by the IIEF, also significantly improved, especially in the 400 mg SAGX intake group. No significant differences were found in PSA levels or testosterone levels. No serious adverse events leading to discontinuation of the study drug were observed in the SAGX groups. **Conclusions:** With fewer side effects than conventional treatments, SAGX is effective and safe in improving symptoms of lower urinary tract symptoms and enhancing erectile function in men.

## 1. Introduction

Lower urinary tract symptoms (LUTSs) represent a constellation of urinary disturbances that significantly impact the quality of life of affected individuals. These symptoms, including urinary frequency, urgency, hesitancy, and nocturia, can stem from a variety of causes, ranging from neurological disorders to lifestyle factors, and are not exclusively related to prostate conditions. The complexity of LUTS etiology necessitates a multifaceted approach to management and treatment [1].

Conventional treatments for LUTSs have primarily focused on pharmacological interventions aimed at alleviating specific symptoms. However, these treatments often come with side effects and may not holistically address the underlying causes of LUTSs. This limitation has led to increased interest in alternative and complementary therapies, especially phytotherapy [2].

Phytotherapy, the use of plant-derived medications in the prevention and treatment of diseases, offers a potential avenue for the management of LUTSs with fewer side effects and a broader approach to treatment. Herbal extracts, such as *saw palmetto*, *red ginseng*, *pygeum africanum*, etc., have been investigated for their effects on urinary tract health with some showing promising results in improving urinary function, reducing inflammation, and enhancing overall urinary tract wellness [3].

*Salvia miltiorrhiza* Bunge, commonly known as Danshen or red sage, is a traditional Chinese medicinal herb that has been used for centuries to treat various ailments, especially in cardiovascular- and cerebrovascular-related disorders, due to its antioxidative stress and antiplatelet aggregation effects [4]. Recent studies have demonstrated the potential of *S. miltiorrhiza root* extract (SAGX) in alleviating BPH symptoms. In vitro experiments have shown that these extracts can inhibit the proliferation of prostate cancer cell lines, suggesting the compound’s natural ability to modulate key signaling pathways presents a promising avenue for non-pharmacological intervention in LUTS management [5].

Furthermore, animal studies have revealed that *S. miltiorrhiza* Bunge extracts can reduce prostate size and improve other markers of prostate health in rat models of BPH [6].

In our current study, we explored the efficacy and safety of a SAGX in alleviating LUTSs.

This multicenter, randomized, double-blind, placebo-controlled clinical trial aims to provide a clearer understanding of SAGX on the male lower urinary tract symptoms and quality of life of men’s health.

## 2. Materials and Methods

### 2.1. Salvia miltiorrhiza Root Extract (SAGX)

The root of *S. miltiorrhiza* was extracted with ethanol, dried, and stored at room temperature in powder form. The production of the *S. miltiorrhiza* capsules was standardized through rigorous quality control processes. Each batch of extract was subjected to High-Performance Liquid Chromatography (HPLC) analysis to confirm consistent levels of key compounds such as salvianolic acid B and dihydrotanshinone I. Furthermore, the extracts were tested for impurities including heavy metals and microbial contaminants, ensuring adherence to safety standards. The test group receiving SAGX was administered two soft capsules, once daily (400 mg or 800 mg per day) for 12 weeks. The placebo group received indistinguishable soft capsules following the same regimen. A venture company developing oriental herbal medicines, the Healthism Corporation (Republic of Korea), developed this product.

### 2.2. Inclusion Criteria

Participants for the human trial were selected based on the following criteria: (1) Men aged 40 to 80 years old. (2) Individuals with an International Prostate Symptom Score (IPSS) of 8 to 19 points (moderate).

### 2.3. Exclusion Criteria

Individuals meeting any of the following conditions were excluded from the human clinical trial:

(1) Persons currently undergoing treatment for severe cardiovascular, immunological, respiratory, hepatobiliary, renal and urological, neurological, musculoskeletal, psychiatric, infectious diseases, or malignant tumors; participation was considered at the investigator’s discretion based on the condition of the clinical trial participant. (2) Individuals with a prostate-specific antigen (PSA) concentration of 3.0 ng/mL or higher, unless a non-cancerous test result was confirmed within 3 months prior to the first visit. (3) Persons with a maximum flow rate of less than 5 mL/s. (4) Individuals with a residual urine volume exceeding 150 mL. (5) Those diagnosed with urolithiasis, urethral stricture, bladder neck contracture, inflammation of the lower urinary tract (bladder, urethra), tuberculosis of the urinary tract, prostatitis, urinary tract infection, acute urinary retention, or neurogenic bladder disorder within 4 weeks prior to the first visit. (6) Persons diagnosed with prostate or bladder cancer. (7) Individuals who underwent prostate-related surgery or other invasive procedures. (8) Persons who received surgeries that could affect lower urinary tract symptoms, such as urethrotomy or bladder neck incision. (9) Patients with uncontrolled hypertension (systolic blood pressure ≥ 160 mmHg or diastolic blood pressure ≥ 100 mmHg, measured after 10 min of rest). (10) Individuals with uncontrolled diabetes (fasting blood glucose ≥ 180 mg/dL or those who started medication for diabetes within the last 3 months). (11) Persons with thyroid disease. (12) Those taking medications for benign prostatic hyperplasia (5-alpha-reductase inhibitors, alpha-blockers, anticholinergics, anti-diuretic hormones, etc.) or prostate health-related dietary supplements (such as those derived from saw palmetto) within 4 weeks prior to the first visit. (13) Individuals with AST (GOT) or ALT (GPT) levels more than three times the upper limit of normal at the trial institution. (14) Persons with creatinine levels more than twice the upper limit of normal at the trial initiation.

### 2.4. Outcome Measurements

The primary endpoint was the change in total IPSS scores (sum of answers to questions 1–7) at the end of treatment compared to baseline. All participants completed IPSS questionnaires at baseline and at the 6th and 12th weeks to compare results before and after treatment among the three groups. The questionnaires are available in the Supplementary Materials, S1 (IPSS) and S2 (IIEF).

The secondary endpoints included changes in the subscores of IPSS for each question as well as changes in PSA, Total Testosterone (Total T), dihydrotestosterone (DHT), maximal urinary flow rate (Qmax), post-void residual volume (PVR), and IIEF, which were measured at baseline and at the 6th and 12th weeks. For Qmax, uroflowmetry was performed using a digital uroflowmeter, which records the flow rate and volume of urine during spontaneous micturition. The participants were instructed to void into the uroflowmeter device, and the highest flow rate achieved during the urination process was recorded.

Safety assessment was evaluated according to adverse events (AEs). Hematology, biochemistry, urinalysis, and vital signs were also observed.

### 2.5. Data Analysis

The within-group comparison of changes in the primary functional evaluation variable, IPSS, before and after intake was analyzed using the paired t-test. The extent of changes between the test group and the control group at each time point was evaluated for statistically significant differences using ANOVA or the Kruskal–Wallis test, depending on whether the assumption of normality was satisfied.

The within-group comparison of changes in the secondary functional evaluation variables, including blood PSA, testosterone (Total Testosterone, free testosterone), DHT, urination speed (maximum and average urination speed), residual urine volume, and IIEF before and after intake, was analyzed using the paired t-test. The extent of changes between the test group and the control group at each time point was evaluated for statistically significant differences using ANOVA or the Kruskal–Wallis test, depending on whether the assumption of normality was satisfied.

When significant differences were found between the test group and the control group, confirmation tests were conducted with ANOVA, and the Wilcoxon rank sum test following the Kruskal–Wallis test. Additionally, a General Linear Model (GLM; identical to ANCOVA) was performed with the baseline values of each functional evaluation variable as covariates.

### 2.6. Reasons for Setting the Intake Amount

Based on the results of previous studies [6], the effective doses of *S. miltiorrhiza root* extract in animal experiments were set at 40 mg/kg and 80 mg/kg. Based on these doses, when applying the Human Equivalent Dose (HED) calculated for a 60 kg body weight, the predicted human dosages of SAGX were approximately 400 mg/60 kg (S-400) and 800 mg/60 kg (S-800). Therefore, the daily intake of SAGX in this human application trial was ultimately set at 400 mg/day and 800 mg/day.

The decision to administer *S. miltiorrhiza root* extract for 12 weeks was based on previous studies of herbal interventions for LUTSs [3].

## 3. Results

### 3.1. Baseline Characteristics and Participant Distribution

The mean ages were 62.75 ± 8.88 (median: 60.5) years in the S-400 group, 62.54 ± 9.25 (median: 61.0) in the S-800 group, and 64.38 ± 9.26 (median 59.5) in the placebo group, showing no statistically significant differences among groups (*p* = 0.5424). Body mass indexes (kg/m^2^) were 24.86 ± 4.95 in S-400, 24.85 ± 6.14 in S-800, and 25.0 ± 5.73 in the placebo group, also showing no significant differences among the groups (*p* = 0.8231).

**Figure 1:**
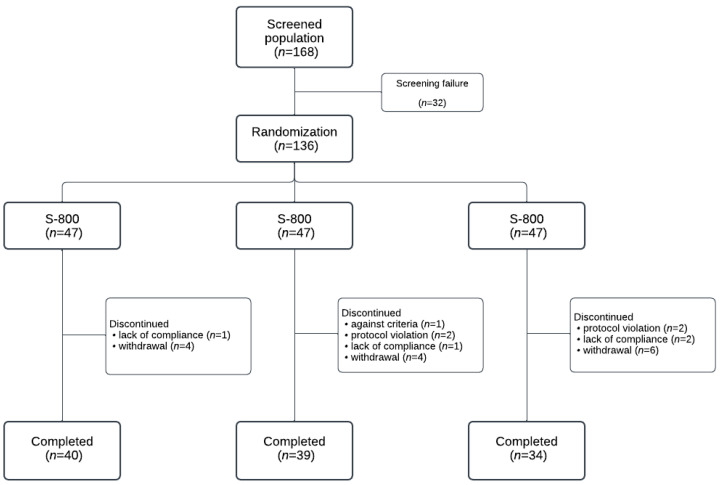
CONSORT diagram of patient disposition.

The initial screening population was 168 individuals, of which 32 were screened out, leaving 136 participants to be randomly assigned. In the S-400 group, 5 of the 45 allocated participants were excluded, allowing 40 participants to complete the trial. In the S-800 group, 8 of the 47 allocated participants were excluded, resulting in 39 participants completing the trial. In the placebo group, 10 of the 44 were excluded, leading to 34 participants completing the trial. The CONSORT diagram is shown in Figure 1.

The clinical trial aimed to assess the efficacy and safety of S-400 and S-800 compared to a placebo for improving lower urinary tract symptoms (LUTSs). A total of 113 participants completed the 12-week study, with 40 in the S-400 group, 39 in the S-800 group, and 34 in the placebo group. The primary outcome measure was a change in the International Prostate Symptom Score (IPSS) from baseline to the study’s conclusion. Secondary outcomes included changes in IPSS subscores and quality of life related to urinary symptoms.

### 3.2. Primary Endpoints

#### 3.2.1. IPSS Total Score

At 12 weeks, both the S-400 and S-800 groups showed a significant reduction in the total IPSS score compared with the placebo group. The mean decrease in the IPSS total score was −4.40 ± 3.14 for the S-400 group and −5.03 ± 4.15 for the S-800 group, compared with −1.06 ± 3.40 in the placebo group (*p* < 0.0001 for both comparisons). This significant improvement indicates a substantial alleviation of LUTSs in participants treated with S-400 and S-800.

#### 3.2.2. IPSS Subscores

Incomplete Emptying

The S-400 and S-800 groups demonstrated significant improvement in the “incomplete emptying” subscore at 12 weeks, with reductions of −0.70 ± 1.16 and −1.13 ± 1.42, respectively, compared with −0.12 ± 1.15 in the placebo group (*p* = 0.0026 and *p* = 0.001, respectively).

Frequency

The “frequency” subscore improvements at 12 weeks were −0.68 ± 0.97 for S-400 and −0.72 ± 1.21 for S-800, which was statistically significant compared to −0.24 ± 0.96 for the placebo group (*p* = 0.0473 and *p* = 0.0135, respectively).

Urgency, Weak Stream, and Nocturia

Improvements in urgency, weak stream, and nocturia subscores were also noted in both treatment groups, with the most significant changes observed in the nocturia subscore, −0.80 ± 0.85 for S-400 and −0.72 ± 0.86 for S-800, versus −0.24 ± 0.70 for the placebo group (*p* = 0.0002 and *p* = 0.001, respectively).

Quality of Life

The quality-of-life score related to urinary symptoms showed significant improvement in the S-400 group (−1.10 ± 0.87) and S-800 group (−0.79 ± 1.00) compared with the placebo group (−0.32 ± 0.84) at the end of 12 weeks (*p* < 0.0001 and *p* = 0.0026, respectively).

The results of primary and secondary outcomes related to IPSS and IPSS subscores are summarized in Table 1.

**Figure 2:**
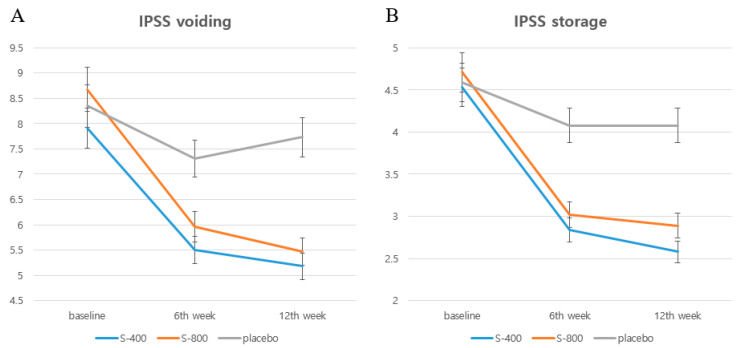
Changes in International Prostate Symptom Score (IPSS) for each group: (**A**) IPSS voiding; (**B**) IPSS storage. Y axis (score).

Figure 2 shows changes in the IPSS voiding subscore (sum of residual urine sensation, intermittency, weak stream, and straining subscores) and the IPSS storage subscore (sum of frequency, urgency, and nocturia subscores).

### 3.3. Secondary Endpoints

#### 3.3.1. International Index of Erectile Function Scores

The IIEF questionnaire, a validated instrument, measures five domains of male sexual function, including erectile function, orgasmic function, sexual desire, intercourse satisfaction, and overall satisfaction. Changes in IIEF scores across each group over the 12-week study period are presented in Table 2.

Total IIEF Score

At baseline, the overall IIEF scores were comparable across all groups. After 12 weeks, the S-400 group experienced a significant improvement in the overall IIEF score (Δ12wk: +9.35 ± 13.64), in contrast to the placebo group, which saw a decrease (−3.35 ± 19.96) resulting in a statistically significant difference (*p* = 0.0006). The S-800 group had a slight increase (Δ12wk: +1.56 ± 17.44), but this did not differ significantly from the placebo group (*p* = 0.1139).

Erectile Function

Significant improvement was observed in the erectile function domain for the S-400 group at 12 weeks (Δ12wk: +5.20 ± 7.50) compared with a decrease in the placebo group (Δ12wk: −1.56 ± 10.89), with a *p*-value of 0.0010. The S-800 group showed marginal improvement (Δ12wk: +0.49 ± 9.51), but the changes were not statistically significant when compared to the placebo group (*p* = 0.1842).

Orgasmic Function, Sexual Desire, and Satisfaction

For orgasmic function and sexual desire, improvements were noted in the S-400 group, with orgasmic function showing a significant increase at 12 weeks (Δ12wk: +1.70 ± 3.05, *p* = 0.0032). Sexual desire improved significantly in both the S-400 (Δ12wk: +0.38 ± 1.66, *p* = 0.0168) and S-800 (Δ12wk: +0.59 ± 1.60, *p* = 0.0031) groups compared with the placebo group.

Interpersonal satisfaction and overall satisfaction also showed improvements in the S-400 group (Δ12wk: +1.50 ± 2.86 for intercourse satisfaction, *p* = 0.0018; Δ12wk: +0.58 ± 1.26 for overall satisfaction, *p* = 0.0029). The S-800 group also had an increase in overall satisfaction (Δ12wk: +0.85 ± 1.27, *p* = 0.0012), indicating a positive trend toward treatment efficacy. Changes in IIEF scores of each group are shown in Table 2.

#### 3.3.2. PSA, Total Testosterone, DHT, Qmax, and PVR

In addition to evaluating the efficacy of S-400 and S-800 in improving lower urinary tract symptoms, we also assessed their impact on various physiological parameters, including PSA, Total T, DHT, Qmax, and PVR. The results for these parameters are summarized in Table 3.

Prostate-Specific Antigen (PSA)

At baseline, PSA levels were comparable across the three groups. After 12 weeks of treatment, there was no statistically significant change in PSA levels among the groups, with a slight increase observed in the placebo group (0.09 ± 0.49 ng/mL) compared to a minor decrease in the S-800 group (−0.26 ± 2.13 ng/mL) and a negligible change in the S-400 group (0.03 ± 0.44 ng/mL) (*p* = 0.8607).

Total Testosterone

Baseline Total Testosterone levels were similar across the groups. After 12 weeks, changes in Total Testosterone levels remained non-significant across the groups, with the S-400 group showing a 0.59 ± 1.42 ng/mL increase, the S-800 group a 0.64 ± 1.99 ng/mL increase, and the placebo group a 0.49 ± 1.56 ng/mL increase (*p* = 0.9115).

Dihydrotestosterone (DHT)

The baseline DHT levels varied among the groups, but the variability was not significant. At the end of the study period, the change in DHT levels did not reach statistical significance, with the S-400 group experiencing an increase of 25.95 ± 91.78 ng/mL and the S-800 group an increase of 9.57 ± 102.68 ng/mL, while the placebo group showed a slight decrease (−1.10 ± 64.27 ng/mL) (*p* = 0.4178).

Maximal Urinary Flow Rate (Qmax)

At baseline, the Qmax was comparable across all groups. After 12 weeks, there were no significant changes in Qmax among the groups. The S-400 group showed a slight increase (0.45 ± 5.28 mL/s), the S-800 group showed a slight decrease (−0.40 ± 7.28 mL/s), and the placebo group remained unchanged (0.00 ± 6.66 mL/s) (*p* = 0.8421).

Post-Void Residual Volume (PVR)

Baseline PVR levels were similar across the groups. After 12 weeks, changes in PVR also did not show significant differences among the groups, with an increase of 4.75 ± 36.93 mL in the S-400 group, 6.46 ± 23.17 mL in the S-800 group, and 1.15 ± 24.94 mL in the placebo group (*p* = 0.6637).

### 3.4. Safety Assessments

Individuals in the S-400 and S-800 groups and placebo groups tolerated treatment well throughout the study period. There were no significant adverse effects reported, including complete blood count (WBC, RBC, Hemoglobin, Platelet, and Eosinophil counts), liver function tests (AST and ALT), kidney function tests (BUN and creatinine), and urinalysis, indicating the safety of these treatments for the management of LUTSs, which underscores the potential of SAGX as a safe alternative for managing erectile dysfunction and other aspects of sexual dysfunction in men.

## 4. Discussion

In a recent preclinical study, *S. miltiorrhiza* extract demonstrated significant efficacy in ameliorating BPH through the regulation of oxidative stress and inflammation. Using a testosterone propionate-induced BPH rat model, the extract (HLT-101) was shown to decrease excessive free radical production and inflammatory factor activation. Additionally, it significantly reduced intracellular reactive oxygen species levels via activation of the Nrf-2/HO-1 signaling pathway in BPH-1 cells. The study also found that HLT-101 inhibited the NF-κB pathway and androgen receptor (AR) signaling, which are closely linked to BPH pathogenesis. These findings suggest that *S. miltiorrhiza* extract can effectively reduce inflammation and oxidative stress, providing a promising alternative treatment approach for BPH [6].

Another in vitro experiment targeting prostate cancer cell lines showed that Cryptotanshinone, a key active component of *S. miltiorrhiza* extract, inhibits androgen receptor (AR) dimerization and the formation of AR–coregulator complexes, leading to the loss of the AR transcriptional activity that induces prostate cell growth. Animal studies with male rat models also revealed a decrease in prostate epithelial thickness and lumen area, along with reduced expression of androgen receptor-related proteins [5].

Alpha-adrenergic blockers, such as terazosin, doxazosin, tamsulosin, alfuzosin, and silodosin, are commonly used as first-line medications for male LUTSs, but it is well known that they can cause side effects such as hypotension or retrograde ejaculation. According to previous meta-analysis studies, alpha-adrenergic blockers have improved total IPSS scores by an average of 3.39 to 7.06 points [7], whereas in this study, after 12 weeks of intake, the S-400 group showed an improvement of 4.40 points, and the S-800 group showed an improvement of 5.03 points, confirming that this is not an inferior treatment. The absence of side effects such as dizziness, hypotension, and headache, which can occur with the use of alpha-adrenergic blockers [8], in the participants of this study suggests the advantage of phytotherapy as a safer alternative for male LUTSs. Specifically, in the case of silodosin, retrograde ejaculation has been shown to occur in about 23% of users [9], which could decrease adherence to medication among sexually active men. No such symptoms were observed in the SAGX intake groups.

5-alpha-reductase inhibitors (5-ARi), such as finasteride or dutasteride, effectively alleviate LUTS symptoms caused by benign hyperplasia, reducing the IPSS total score by approximately 4.1 points [10]. However, their use may be limited due to reports of potential side effects such as erectile dysfunction and decreased libido [11]. In contrast, SAGX does not have these side effects, making it a more viable option for sexually active men. Additionally, unlike 5-ARi, which can alter serum PSA and testosterone levels posing a risk of masking prostate cancer, SAGX does not have this drawback.

Compared to *Serenoa repens*, known for reducing IPSS total scores by 3.0 to 3.2 points in treating male LUTSs, SAGX has demonstrated superior symptom improvement [12].

Additionally, the IIEF total score significantly improved by the 12th week of S-400 administration, suggesting increased efficacy with long-term use. Conversely, saw palmetto has not produced significant changes in IIEF-5 symptom scores, highlighting SAGX’s potential advantages in enhancing sexual function alongside LUTS management [13].

This study’s limitations include not measuring prostate size via transrectal ultrasonography due to participant discomfort and recruitment convenience. Also, although reductions in Qmax and PVR were observed in the SAGX treatment groups, they were not statistically significant. Similar to previous phytotherapy research, while IPSS scores improved, enhancements in uroflowmetry were not confirmed [14]. However, the significant IPSS results observed within the study’s short 12-week duration suggest the potential for meaningful uroflowmetry outcomes with longer-term treatment.

Additionally, a clear dose-dependent response between the 400 mg and 800 mg SAGX groups was not consistently observed; there were specific outcomes where the 800 mg group demonstrated superior results compared to the 400 mg group. For example, in the improvement in the IPSS subscores related to “frequency” and “incomplete emptying”, as well as in the quality-of-life metrics, the 800 mg group tended to show greater mean changes compared to the 400 mg group, although these differences did not reach statistical significance.

To the best of our knowledge, this is the first clinical trial applying *S. miltiorrhiza* extract to male lower urinary tract symptoms (LUTSs), aiming to pave the way for more personalized and holistic treatment options. This research contributes valuable insights into the potential of phytotherapy as a complementary or alternative strategy for individuals experiencing LUTSs, highlighting its significance in advancing care in this field.

## 5. Conclusions

The data from this study demonstrate that SAGX significantly improves symptoms in men with LUTSs, as evidenced in total IPSS, and also showed positive trends in several domains of male sexual function, including sexual desire and overall satisfaction, as measured by the IIEF scores. These findings highlight the potential of SAGX as an effective treatment for enhancing male sexual function as well as LUTSs. Further research is needed to fully understand the mechanisms behind these improvements and to confirm the long-term efficacy and safety of SAGX in a larger population.

## Figures and Tables

**Table 1:** Changes in IPSS total score and IPSS subscores of each group.

	S-400 (N = 40)	S-800 (N = 39)	Placebo (P) (N = 34)	*p*-Value *	
				S-400 vs. P ^W^	S-800 vs. P ^W^
**IPSS total ^B^**	12.43 ± 2.88	13.41 ± 3.45	12.94 ± 2.93	0.4229	
Δ6wk	−3.70 ± 2.90	−4.41 ± 3.21	−1.65 ± 3.45	0.0065	0.0009
Δ12wk	−4.40 ± 3.14	−5.03 ± 4.15	−1.06 ± 3.40	<0.0001	<0.0001
**Incomplete emptying ^B^**	1.93 ± 1.05	2.38 ± 1.29	2.12 ± 1.17	0.4184	
Δ6wk	−0.70 ± 0.94	−0.95 ± 1.10	−0.15 ± 1.02	0.001	0.0017
Δ12wk	−0.70 ± 1.16	−1.13 ± 1.42	−0.12 ± 1.15	0.0026	0.001
**Frequency ^B^**	1.68 ± 1.02	1.51 ± 1.25	1.56 ± 1.28	0.6697	
Δ6wk	−0.38 ± 0.90	−0.59 ± 1.02	−0.35 ± 0.77	0.9033	0.1302
Δ12wk	−0.68 ± 0.97	−0.72 ± 1.21	−0.24 ± 0.96	0.0473	0.0135
**Intermittency ^B^**	2.05 ± 1.13	2.41 ± 1.41	2.15 ± 1.42	0.5514	
Δ6wk	−0.73 ± 0.82	−0.82 ± 1.32	−0.24 ± 1.07	0.0066	0.0514
Δ12wk	−0.75 ± 1.17	−1.00 ± 1.30	−0.18 ± 1.06	0.0017	0.0017
**Urgency ^B^**	0.85 ± 0.80	1.28 ± 1.23	0.91 ± 1.16	0.2396	
Δ6wk	−0.08 ± 0.69	−0.46 ± 0.82	0.06 ± 0.85	0.3878	0.0263
Δ12wk	−0.20 ± 0.76	−0.38 ± 1.18	0.03 ± 0.94	0.1619	0.2917
**Weak stream ^B^**	2.63 ± 1.19	2.56 ± 1.10	2.79 ± 1.20	0.4045	
Δ6wk	−0.68 ± 1.07	−0.41 ± 0.97	−0.18 ± 1.42	0.0305	0.1481
Δ12wk	−0.65 ± 1.10	−0.44 ± 1.10	0.00 ± 1.35	0.001	0.006
**Straining ^B^**	1.30 ± 1.11	1.33 ± 0.98	1.29 ± 1.12	0.9394	
Δ6wk	−0.30 ± 0.97	−0.54 ± 0.82	−0.47 ± 1.16	0.3818	0.8045
Δ12wk	−0.63 ± 0.90	−0.64 ± 0.90	−0.32 ± 0.81	0.0711	0.0866
**Nocturia ^B^**	2.00 ± 1.13	1.92 ± 0.93	2.12 ± 0.88	0.5925	
Δ6wk	−0.85 ± 0.86	−0.64 ± 0.87	−0.32 ± 0.91	0.0021	0.0146
Δ12wk	−0.80 ± 0.85	−0.72 ± 0.86	−0.24 ± 0.70	0.0002	0.001
**Quality of life ^B^**	3.70 ± 0.69	3.59 ± 0.99	3.79 ± 0.54	0.9149	
Δ6wk	−0.75 ± 0.90	−0.56 ± 1.05	−0.38 ± 0.95	0.037	0.0962
Δ12wk	−1.10 ± 0.87	−0.79 ± 1.00	−0.32 ± 0.84	<0.0001	0.0026

Δ = Score changes from the baseline. Data are presented as mean ± standard deviation. ^B^ Baseline; * compared among groups (Kruskal–Wallis test or ANOVA); and ^W^ compared between two groups (Wilcoxon rank sum test, GLM adjusted baseline).

**Table 2:** Changes in the IIEF scores of each group.

	S-400 (N = 40)	S-800 (N = 39)	Placebo (P) (N = 34)	*p*-Value *	
				S-400 vs. P ^W^	S-800 vs. P ^W^
**IIEF total ^B^**	31.80 ± 20.18	34.92 ± 19.02	30.44 ± 19.22	0.8176	
Δ6wk	3.50 ± 13.83	3.31 ± 18.47	1.15 ± 12.25	0.4159	0.3620
Δ12wk	9.35 ± 13.64	1.56 ± 17.44	−3.35 ± 19.96	0.0006	0.1139
**Erectile function ^B^**	12.00 ± 10.14	14.10 ± 9.56	11.82 ± 9.83	0.8877	
Δ6wk	2.68 ± 7.47	1.21 ± 10.03	0.53 ± 5.90	0.1736	0.4806
Δ12wk	5.20 ± 7.50	0.49 ± 9.51	−1.56 ± 10.89	0.0010	0.1842
**Orgasmic function ^B^**	3.85 ± 3.99	4.44 ± 3.73	3.47 ± 3.65	0.5844	
Δ6wk	0.48 ± 2.86	0.41 ± 3.97	0.38 ± 2.72	0.7910	0.6700
Δ12wk	1.70 ± 3.05	−0.05 ± 3.70	−0.47 ± 4.05	0.0032	0.3123
**Sexual desire ^B^**	6.30 ± 1.68	6.10 ± 1.77	6.09 ± 1.60	0.7935	
Δ6wk	−0.23 ± 1.27	0.72 ± 1.32	−0.12 ± 1.15	0.7281	0.0031
Δ12wk	0.38 ± 1.66	0.59 ± 1.60	−0.35 ± 1.39	0.0168	0.0031
**Intercourse satisfaction ^B^**	4.08 ± 4.23	5.08 ± 4.11	3.88 ± 3.96	0.5749	
Δ6wk	0.60 ± 2.70	0.33 ± 3.85	0.35 ± 2.63	0.6479	0.7149
Δ12wk	1.50 ± 2.86	−0.31 ± 3.68	−0.82 ± 4.27	0.0018	0.2288
**Overall satisfaction ^B^**	5.58 ± 1.66	5.21 ± 1.44	5.18 ± 1.73	0.5328	
Δ6wk	−0.03 ± 1.42	0.64 ± 1.16	0.00 ± 1.63	0.7307	0.0361
Δ12wk	0.58 ± 1.26	0.85 ± 1.27	−0.15 ± 1.42	0.0029	0.0012

Δ = Score changes from the baseline. Data are presented as mean ± standard deviation. ^B^ Baseline; * compared among groups (Kruskal–Wallis test or ANOVA); and ^W^ compared between two groups (Wilcoxon rank sum test, GLM adjusted baseline).

**Table 3:** Changes in PSA, Total Testosterone, DHT, Qmax, and PVR of each group.

	S-400 (N = 40)	S-800 (N = 39)	Placebo (P) (N = 34)	*p*-Value *
**PSA ^B^ (ng/mL)**	1.15 ± 0.73	1.74 ± 3.00	1.08 ± 0.54	0.7171
**Δ12wk**	0.03 ± 0.44	−0.26 ± 2.13	0.09 ± 0.49	0.8607
**Total T ^B^ (ng/mL)**	3.93 ± 1.39	4.63 ± 2.12	4.34 ± 1.76	0.5692
**Δ12wk**	0.59 ± 1.42	0.64 ± 1.99	0.49 ± 1.56	0.9115
**DHT ^B^ (ng/mL)**	277.62 ± 96.23	335.65 ± 127.08	308.82 ± 107.04	0.1288
**Δ12wk**	25.95 ± 91.78	9.57 ± 102.68	−1.10 ± 64.27	0.4178
**Qmax ^B^ (mL/s)**	13.30 ± 7.25	15.11 ± 7.41	12.45 ± 5.99	0.2064
**Δ12wk**	0.45 ± 5.28	−0.40 ± 7.28	0.00 ± 6.66	0.8421
**PVR ^B^ (mL)**	20.88 ± 30.14	15.41 ± 16.97	19.68 ± 23.64	0.9227
**Δ12wk**	4.75 ± 36.93	6.46 ± 23.17	1.15 ± 24.94	0.6637

PSA (prostate-specific antigen), Total T (Total Testosterone), DHT (dihydrotestosterone), Qmax (maximal urinary flow rate), and PVR (post-void residual volume). Δ = Score changes from the baseline. Data are presented as mean ± standard deviation. ^B^ Baseline; * compared among groups (Kruskal–Wallis test or ANOVA).

## Data Availability

The data presented in this paper are available upon request from the corresponding author. The data are not publicly available due to privacy reasons.

## Supplementary Materials

The following supporting information can be downloaded at: https://www.mdpi.com/article/10.3390/nu17010024/s1, S1: IPSS, S2: IIEF [15].
