# Supplementary material for: Salvia miltiorrhiza Root Extract for Men with Lower Urinary Tract Symptoms: A Multicenter, Randomized, Double-Blind, Placebo-Controlled Trial
Source: Nutrients. 2024 Dec 25;17(1):24. doi: 10.3390/nu17010024 (PMC11723278; doi:10.3390/nu17010024)
Supplement: Supplementary file 1 [file nutrients-17-00024-s001.zip › supplementary1_IPSS.pdf]

INTERNATIONAL PROSTATE SYMPTOM SCORE (I-PSS)

Patient Name:<br><br>Date:	Not At All	Less Than 1 Time In 5	Less Than Half The Time	About Half The Time	More Than Half The Time	Almost Always	YOUR SCORE
**1. Incomplete Emptying**<br>Over the past month, how often have you had a sensation of not emptying your bladder completely after you finish urinating?	0	1	2	3	4	5	
**2. Frequency**<br>Over the past month, how often have you had to urinate again less than two hours after you have finished urinating?	0	1	2	3	4	5	
**3. Intermittency**<br>Over the past month, how often have you found you stopped and started again several times when you urinated?	0	1	2	3	4	5	
**4. Urgency**<br>Over the past month, how often have you found it difficult to postpone urination?	0	1	2	3	4	5	
**5. Weak Stream**<br>Over the last month, how often have you had a weak urinary stream?	0	1	2	3	4	5	
**6. Straining**<br>Over the past month, how often have you had to push or strain to begin urination?	0	1	2	3	4	5	
	**None**	**Once**	**Twice**	**3 times**	**4 times**	**5 or more**	**YOUR SCORE**
**7. Nocturia**<br>Over the past month how many times did you most typically get up each night to urinate from the time you went to bed until the time you got up in the morning?	0	1	2	3	4	5	
**Total I-PSS Score**							

**Quality of Life due to Urinary Symptoms**	Delighted	Pleased	Mostly satisfied	Mixed	Mostly unhappy	Unhappy	Terrible
If you were to spend the rest of your life with your urinary condition just the way it is now, how would you feel about that?	0	1	2	3	4	5	6

The I-PSS is based on the answers to seven questions concerning urinary symptoms. Each question is assigned points from 0 to 5 indicating increasing severity of the particular symptom. The total score can therefore range from 0 to 35 (asymptomatic to very symptomatic).

Although there are presently no standard recommendations into grading patients with mild, moderate or severe symptoms, patients can be tentatively classified as follows: **0 - 7 = mildly symptomatic; 8 - 19 = moderately symptomatic; 20 - 35 = severely symptomatic.**

The International Consensus Committee (ICC) recommends the use of only a single question to assess the patient's quality of life. The answers to this question range from "delighted" to "terrible" or 0 to 6. Although this single question may or may not capture the global impact of BPH symptoms on quality of life, it may serve as a valuable starting point for doctor-patient conversation.
