# Supplementary material for: Salvia miltiorrhiza Root Extract for Men with Lower Urinary Tract Symptoms: A Multicenter, Randomized, Double-Blind, Placebo-Controlled Trial
Source: Nutrients. 2024 Dec 25;17(1):24. doi: 10.3390/nu17010024 (PMC11723278; doi:10.3390/nu17010024)
Supplement: Supplementary file 1 [file nutrients-17-00024-s001.zip › supplementary2_IIEF.pdf]

INTERNATIONAL INDEX OF ERECTILE FUNCTION

Patient Questionnaire

HOSPITAL NUMBER (IF KNOWN) ☐☐☐☐☐☐☐

NAME ……………………………………………………………………

DATE OF BIRTH ☐☐ ☐☐ ☐☐☐☐ AGE ☐☐

ADDRESS ……………………………………………………………………
……………………………………………………………………
……………………………………………………………………

TELEPHONE ……………………………………………………………………

These questions ask about the effects that your erection problems have had on your sex life <u>over the last four weeks</u>. Please try to answer the questions as honestly and as clearly as you are able. Your answers will help your doctor to choose the most effective treatment suited to your condition. In answering the questions, the following definitions apply:

- **sexual activity** includes intercourse, caressing, foreplay & masturbation
- **sexual intercourse** is defined as sexual penetration of your partner
- **sexual stimulation** includes situation such as foreplay, erotic pictures etc.
- **ejaculation** is the ejection of semen from the penis (or the feeling of this)
- **orgasm** is the fulfilment or climax following sexual stimulation or intercourse

---

OVER THE PAST 4 WEEKS
CHECK ONE BOX ONLY

☐ **Q1**	**How often were you able to get an erection during sexual activity?**	0 No sexual activity<br>1 Almost never or never<br>2 A few times (less than half the time)<br>3 Sometimes (about half the time)<br>4 Most times (more than half the time)<br>5 Almost always or always
☐ **Q2**	**When you had erections with sexual stimulation, how often were your erections hard enough for penetration?**	0 No sexual activity<br>1 Almost never or never<br>2 A few times (less than half the time)<br>3 Sometimes (about half the time)<br>4 Most times (more than half the time)<br>5 Almost always or always
☐ **Q3**	**When you attempted intercourse, how often were you able to penetrate (enter) your partner?**	0 Did not attempt intercourse<br>1 Almost never or never<br>2 A few times (less than half the time)<br>3 Sometimes (about half the time)<br>4 Most times (more than half the time)<br>5 Almost always or always
☐ **Q4**	**During sexual intercourse, <u>how often</u> were you able to maintain your erection after you had penetrated (entered) your partner?**	0 Did not attempt intercourse<br>1 Almost never or never<br>2 A few times (less than half the time)<br>3 Sometimes (about half the time)<br>4 Most times (more than half the time)<br>5 Almost always or always
☐ **Q5**	**During sexual intercourse, <u>how difficult</u> was it to maintain your erection to completion of intercourse?**	0 Did not attempt intercourse<br>1 Extremely difficult<br>2 Very difficult<br>3 Difficult<br>4 Slightly difficult<br>5 Not difficult

☐	Q6	**How many times have you attempted sexual intercourse?**	0 No attempts<br>1 One to two attempts<br>2 Three to four attempts<br>3 Five to six attempts<br>4 Seven to ten attempts<br>5 Eleven or more attempts
☐	Q7	**When you attempted sexual intercourse, how often was it satisfactory for you?**	0 Did not attempt intercourse<br>1 Almost never or never<br>2 A few times (less than half the time)<br>3 Sometimes (about half the time)<br>4 Most times (more than half the time)<br>5 Almost always or always
☐	Q8	**How much have you enjoyed sexual intercourse?**	0 No intercourse<br>1 No enjoyment at all<br>2 Not very enjoyable<br>3 Fairly enjoyable<br>4 Highly enjoyable<br>5 Very highly enjoyable
☐	Q9	**When you had sexual stimulation <u>or</u> intercourse, how often did you ejaculate?**	0 No sexual stimulation or intercourse<br>1 Almost never or never<br>2 A few times (less than half the time)<br>3 Sometimes (about half the time)<br>4 Most times (more than half the time)<br>5 Almost always or always
☐	Q10	**When you had sexual stimulation <u>or</u> intercourse, how often did you have the feeling of orgasm or climax?**	1 Almost never or never<br>2 A few times (less than half the time)<br>3 Sometimes (about half the time)<br>4 Most times (more than half the time)<br>5 Almost always or always
☐	Q11	**How often have you felt sexual desire?**	1 Almost never or never<br>2 A few times (less than half the time)<br>3 Sometimes (about half the time)<br>4 Most times (more than half the time)<br>5 Almost always or always
☐	Q12	**How would you rate your level of sexual desire?**	1 Very low or none at all<br>2 Low<br>3 Moderate<br>4 High<br>5 Very high
☐	Q13	**How satisfied have you been with your <u>overall sex life</u>?**	1 Very dissatisfied<br>2 Moderately dissatisfied<br>3 Equally satisfied & dissatisfied<br>4 Moderately satisfied<br>5 Very satisfied
☐	Q14	**How satisfied have you been with your <u>sexual relationship</u> with your partner?**	1 Very dissatisfied<br>2 Moderately dissatisfied<br>3 Equally satisfied & dissatisfied<br>4 Moderately satisfied<br>5 Very satisfied
☐	Q15	**How do you rate your <u>confidence</u> that you could get and keep an erection?**	1 Very low<br>2 Low<br>3 Moderate<br>4 High<br>5 Very high

INTERNATIONAL INDEX OF ERECTILE FUNCTION (IIEF)

Guidelines on Clinical Application of IIEF Patient Questionnaire

Background

The 15-question International Index of Erectile Function (IIEF) Questionnaire is a validated, multi-dimensional, self-administered investigation that has been found useful in the clinical assessment of erectile dysfunction and treatment outcomes in clinical trials. A score of 0-5 is awarded to each of the 15 questions that examine the 4 main domains of male sexual function: erectile function, orgasmic function, sexual desire and intercourse satisfaction.

In a recent study[1],the IIEF Questionnaire was tested in a series of 111 men with sexual dysfunction and 109 age-matched, normal volunteers. The following mean scores were recorded:

FUNCTION DOMAIN	MAX SCORE	CONTROLS	PATIENTS
**A. Erectile Function (Q1,2,3,4,5,15)**	30	25.8	10.7
**B. Orgasmic Function (Q9,10)**	10	9.8	5.3
**C. Sexual Desire (Q11,12)**	10	7.0	6.3
**D. Intercourse Satisfaction (Q6,7,8)**	15	10.6	5.5
**E. Overall Satisfaction (Q13,14)**	10	8.6	4.4

Clinical Application

IIEF assessment is limited by the superficial assessment of psychosexual background and the very limited assessment of partner relationship, both important factors in the presentation of male sexual dysfunction. Analysis of the questionnaire should, therefore, be viewed as an adjunct to, rather than a substitute for, a detailed sexual history and examination. The following guide-lines may be applied:

1. Patients with low IEEF scores (<14 out of 30) in Domain A (Erectile Function) may be considered for a trial course of therapy with Sildenafil unless contraindicated. Specialist referral is indicated if this is unsuccessful.

2. Patients demonstrating primary orgasmic or ejaculatory dysfunction (Domain B) should be referred for specialist investigation.

3. Patients with reduced sexual desire (Domain C) require testing of blood levels of androgen and prolactin.

4. Psychosexual counselling should be considered if low scores are recorded in Domains D and E but there is only a moderately lowered score (14 to 25) in Domain A.

Reference

1. Rosen R, Riley A, Wagner G, et al. The International Index of Erectile Function (IIEF): A multidimensional scale for assessment of erectile dysfunction. *Urology*, 1997, **49**: 822-830.
